# Who did Buzz see someone? Grammaticality judgement of wh-questions in typically developing children and children with Grammatical-SLI

**DOI:** 10.1016/j.lingua.2010.10.007

**Published:** 2011-02

**Authors:** Heather K.J. van der Lely, Melanie Jones, Chloë R. Marshall

**Affiliations:** Centre for Developmental Language Disorders and Cognitive Neuroscience, DLDCN.org, London, United Kingdom

**Keywords:** Language acquisition, G-SLI, Grammaticality judgement, Wh-questions

## Abstract

This paper tests claims that children with Grammatical(G)-SLI are impaired in hierarchical structural dependencies at the clause level and in whatever underlies such dependencies with respect to movement, chain formation and feature checking; that is, their impairment lies in the syntactic computational system itself (the Computational Grammatical Complexity hypothesis proposed by van der Lely in previous work). We use a grammaticality judgement task to test whether G-SLI children's errors in wh-questions are due to the hypothesised impairment in syntactic dependencies at the clause level or lie in more general processes outside the syntactic system, such as working memory capacity. We compare the performance of 14 G-SLI children (aged 10–17 years) with that of 36 younger language-matched controls (aged 5–8 years). We presented matrix wh-subject and object questions balanced for wh-words (*who/what/which*) that were grammatical, ungrammatical, or semantically inappropriate. Ungrammatical questions contained wh-trace or T-to-C dependency violations that G-SLI children had previously produced in elicitation tasks. G-SLI children, like their language controls, correctly accepted grammatical questions, but rejected semantically inappropriate ones. However, they were significantly impaired in rejecting wh-trace and T-to-C dependency violations. The findings provide further support for the CGC hypothesis that G-SLI children have a core deficit in the computational system itself that affects syntactic dependencies at the clause level.
